# Single-cell transcriptome sequencing reveals that *Wolbachia* induces gene expression changes in *Drosophila* ovary cells to favor its own maternal transmission

**DOI:** 10.1128/mbio.01473-24

**Published:** 2024-08-28

**Authors:** Yun-heng Miao, Wei-hao Dou, Jing Liu, Da-wei Huang, Jin-hua Xiao

**Affiliations:** 1 College of Life Sciences, Nankai University, Tianjin, China; University of Hawaii at Manoa, Honolulu, Hawaii, USA; University of Hawaii at Manoa, Honolulu, Hawaii, USA

**Keywords:** single-cell mRNA sequencing, *Wolbachia*, *Drosophila *ovary, maternal transmission

## Abstract

*Wolbachia* is an obligate endosymbiont that is maternally inherited and widely distributed in arthropods and nematodes. It remains in the mature eggs of female hosts over generations through multiple strategies and manipulates the reproduction system of the host to enhance its spreading efficiency. However, the transmission of *Wolbachia* within the host’s ovaries and its effects on ovarian cells during oogenesis, have not been extensively studied. We used single-cell RNA sequencing to comparatively analyze cell-typing and gene expression in *Drosophila* ovaries infected and uninfected with *Wolbachia*. Our findings indicate that *Wolbachia* significantly affects the transcription of host genes involved in the extracellular matrix, cytoskeleton organization, and cytomembrane mobility in multiple cell types, which may make host ovarian cells more conducive for the transmission of *Wolbachia* from extracellular to intracellular. Moreover, the genes *nos* and *orb*, which are related to the synthesis of ribonucleoprotein complexes, are specifically upregulated in early germline cells of ovaries infected with *Wolbachia*, revealing that *Wolbachia* can increase the possibility of its localization to the host oocytes by enhancing the binding with host ribonucleoprotein-complex processing bodies (P-bodies). All these findings provide novel insights into the maternal transmission of *Wolbachia* between host ovarian cells.

**IMPORTANCE:**

*Wolbachia*, an obligate endosymbiont in arthropods, can manipulate the reproduction system of the host to enhance its maternal transmission and reside in the host’s eggs for generations. Herein, we performed single-cell RNA sequencing of ovaries from *Drosophila melanogaster* and observed the effects of *Wolbachia* (strain *w*Mel) infection on different cell types to discuss the potential mechanism associated with the transmission and retention of *Wolbachia* within the ovaries of female hosts. It was found that the transcriptions of multiple genes in the ovary samples infected with *Wolbachia* are significantly altered, which possibly favors the maternal transmission of *Wolbachia*. Meanwhile, we also discovered that *Wolbachia* may flexibly regulate the expression level of specific host genes according to their needs rather than rigidly changing the expression level in one direction to achieve a more suitable living environment in the host’s ovarian cells. Our findings contribute to a further understanding of the maternal transmission and possible universal effects of *Wolbachia* within the host.

## INTRODUCTION

*Wolbachia* is a maternally inherited intracellular bacterium widely distributed in arthropods and nematodes. A key feature of *Wolbachia* is the ability to affect the biological processes of its host, such as behavior, metabolism, immunity, and reproduction (1). *Wolbachia* manipulates the host’s reproduction to enhance its own maternal transmission, mainly at two levels: (i) entering the germ cells of female hosts, residing in the mature oocytes, and (ii) creating a more favorable environment in the host offspring for *Wolbachia*.

*Wolbachia* employs various strategies to facilitate its transfer and retention in the germ cells of female hosts. First, the distribution of *Wolbachia* in the host is tissue-biased, as it is mainly found in the vicinity of the ovaries (2). In *Drosophila* ovaries, *Wolbachia* always has the pattern of somatic stem cell niche (SSCN) or germline stem cell niche (GSCN) tropism, which means that it initially accumulates in stem cells and later spreads to other cell types. This tropism depends on the *Wolbachia* strains rather than on the hosts (3, 4). During oogenesis, the SSCN develops into follicle cells surrounding the germline and is responsible for eggshell generation when the oocyte matures. The GSCN consists of terminal filament (TF), cap cell (CC), and germline stem cell (GSC). GSC divides asymmetrically into daughter cells. The daughter cell then undergoes four mitotic divisions into a 16-cell cyst, only one cell of which will differentiate into the oocyte, while the others become nurse cells, synthesizing nutrients and cytoplasmic components for the oocyte (3–5). Second, *Wolbachia* enhances the female host’s GSC proliferation after entering the germarium, which can increase the number of host offspring to promote its spreading efficiency (6–8). Third, the *Wolbachia* titer is also important for its maternal transmission, which is related to various factors in the host cellular environment. For instance, *Wolbachia* titer is positively correlated with mitochondrial density and autophagy intensity (9, 10) while negatively regulated by *Wolbachia density suppressor* (*Wds*) (11). Importantly, actin in the host cells is critical for efficient maternal transmission of *Wolbachia. Wolbachia* is not easily retained in the offspring of fruit flies with heterozygous mutations in the cytoskeletal proteins profilin and villin, probably because the maternal transmission of *Wolbachia* is dependent on the combination of its own secreted protein WD0830 and the host actin (12, 13).

In arthropods, *Wolbachia* induces several reproductive manipulations to make the intracellular environment of the host offspring more suitable for its living, mainly by increasing the number of infected females in the offspring. Cytoplasmic incompatibility (CI) is the most extensively studied, in which *Wolbachia*-infected males mating with *Wolbachia*-uninfected females produce embryos that fail to survive due to abnormal deposition of histone His3.3 in male pronucleus, delayed activation of the cell cycle kinase Cdk1 and nuclear envelope breakdown prior to the first mitosis, resulting in chromosome condensation and abnormal cell division (14–16). The earliest proposed hypothesis has suggested that CI involves the modification of sperm by a *Wolbachia* factor, which can be rescued by another factor from *Wolbachia*-infected eggs. Subsequently, it has been suggested that *Wolbachia*-encoded CI factors (Cifs) are critical for the induction and rescue of CI. Some prominent mechanism models have been proposed, such as the Host-Modification (HM) and Toxin-Antidote (TA) models (17). The HM model suggests that Cif proteins directly modify sperm, leading to the failure of male pronucleus chromosome segregation in the embryo, unless this modification is reversed or removed by CifA derived from *Wolbachia*-infected eggs. The TA model suggests that, in the embryo, CifB in male gamete plays a toxin role leading to CI, while maternally sourced CifA in the embryo is an antidote that removes the CI effect. Several in-depth studies have supported these two models to some extent, such as the discovery of sperm-derived CifB in fertilized embryos, and the process of CI rescue relying on the binding of CifA to sperm-derived CifB, thus demonstrating that CifB can enter fertilized embryos (18–20). Multiple omics studies have also shown that *Wolbachia* exerts CI-related effects on multiple host genes and metabolic pathways in the male testis, implying the modification role of *Wolbachia* in spermatogenesis (21–29).

Nonetheless, the mechanisms underlying the transmission and retention of *Wolbachia* within the ovaries of female hosts remain elusive. Although certain host factors that potentially influence *Wolbachia* titer and its maternal transmission have been identified, how *Wolbachia* transmits from stem cells to the whole ovarian cells during oogenesis and how *Wolbachia* steadily localizes in the mature oocytes have not been investigated, likely due to limited knowledge regarding the changes of gene expression in ovarian cells at different developmental stages after *Wolbachia* infection. Therefore, we conducted a single-cell RNA sequencing analysis on *Wolbachia*-infected and -uninfected *Drosophila melanogaster* ovaries, focusing on the key genes that *Wolbachia* highly influences. We compared the effects of *Wolbachia* on gene transcription in different ovarian cell types and found their potential associations with the maternal transmission of *Wolbachia*.

## RESULTS

### Cell type identification

**Fig 1:**
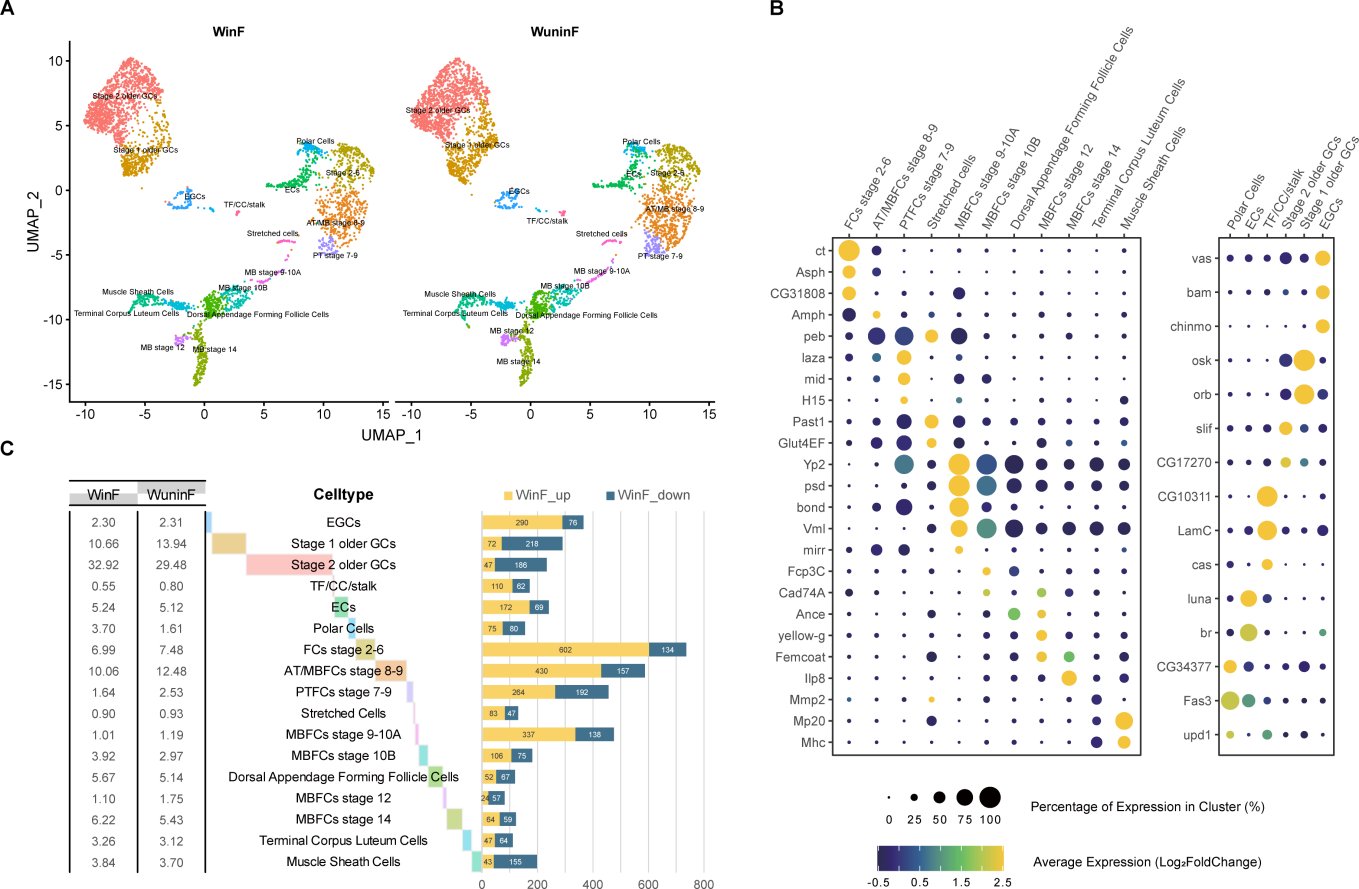
scRNA-seq of *Drosophila* adult ovary samples. (**A**) UMAP plot showed the 17 major cell types identified from the *Drosophila* ovary and was colored according to cluster membership. (**B**) Dot plot of scaled expression of marker genes in each cell type. (**C**) Basic statistics of cell types in WinF and WuninF. The proportion of the cell number of each cell type was counted and listed on the left, and the lengths of transparent bars behind cell types displayed the mean proportion between WinF and WuninF samples. Bars on the right represented the DEG numbers [the genes could be detected in ≥25% cells in both WinF and WuninF cluster populations, |log2FC| ≥0.25 and *P* value < 0.05 (Wilcox *t* test)] when WinF was compared to WuninF. The upregulated genes in WinF were colored yellow and those downregulated in WinF were colored blue.

To investigate the influence of *Wolbachia* on the gene expression of diverse cell types in the *Drosophila* ovaries, we performed single-cell RNA sequencing for ovary samples infected with *Wolbachia* (WinF) and those uninfected (WuninF). After quality control, 3,648 and 4,104 cells were obtained in WinF and WuninF, respectively. We identified 17 cell types (14 somatic cell types and 3 germ cell types) in both WinF and WuninF (Fig. 1A) using the published marker genes of different *Drosophila* ovary cell types as references (30–32) (Fig. 1B) (TF/CC/stalk: Terminal Filament/Cap Cells/stalk cells; EGCs: Early Germline Cells; GC: Germ cells; ATFC: Anterior terminal follicle cells; MBFC: Mainbody follicle cells; PTFC: Posterior terminal follicle cells). Among the three germ cell types, the EGCs cluster included the cells in Region 1 (before the 16-cell cyst) of the germarium, and the Stage 1 older GCs cluster included the cells from Region 2 of the germarium and onward (30). Stage 2 older GCs cluster represented the germ cells in Stage 2 follicle (32). We determined the proportion of cell types in both samples and found that the clustering patterns of the cell types in WinF and WuninF were similar (Fig. 1C). Then we identified the differentially expressed genes (DEGs) of each cell type between WinF and WuninF with the regular parameters of |log2FC| ≥0.25 and *P* value < 0.05 (Wilcox *t* test). Additionally, we applied an advanced filter to select genes detected in at least 25% of cells in both WinF and WuninF cluster populations. Most DEGs were identified in germ cells and early- and middle-stage follicle cells. By contrast, fewer DEGs were found in MBFCs Stage 12 and MBFCs Stage 14 (Table S1; Fig. 1C), indicating that *Wolbachia* exerts greater effects on the gene expression in the early- and middle-developing stages of *Drosophila* ovary.

### Global effects of *Wolbachia* in egg membrane formation of ovarian cells

**Fig 2:**
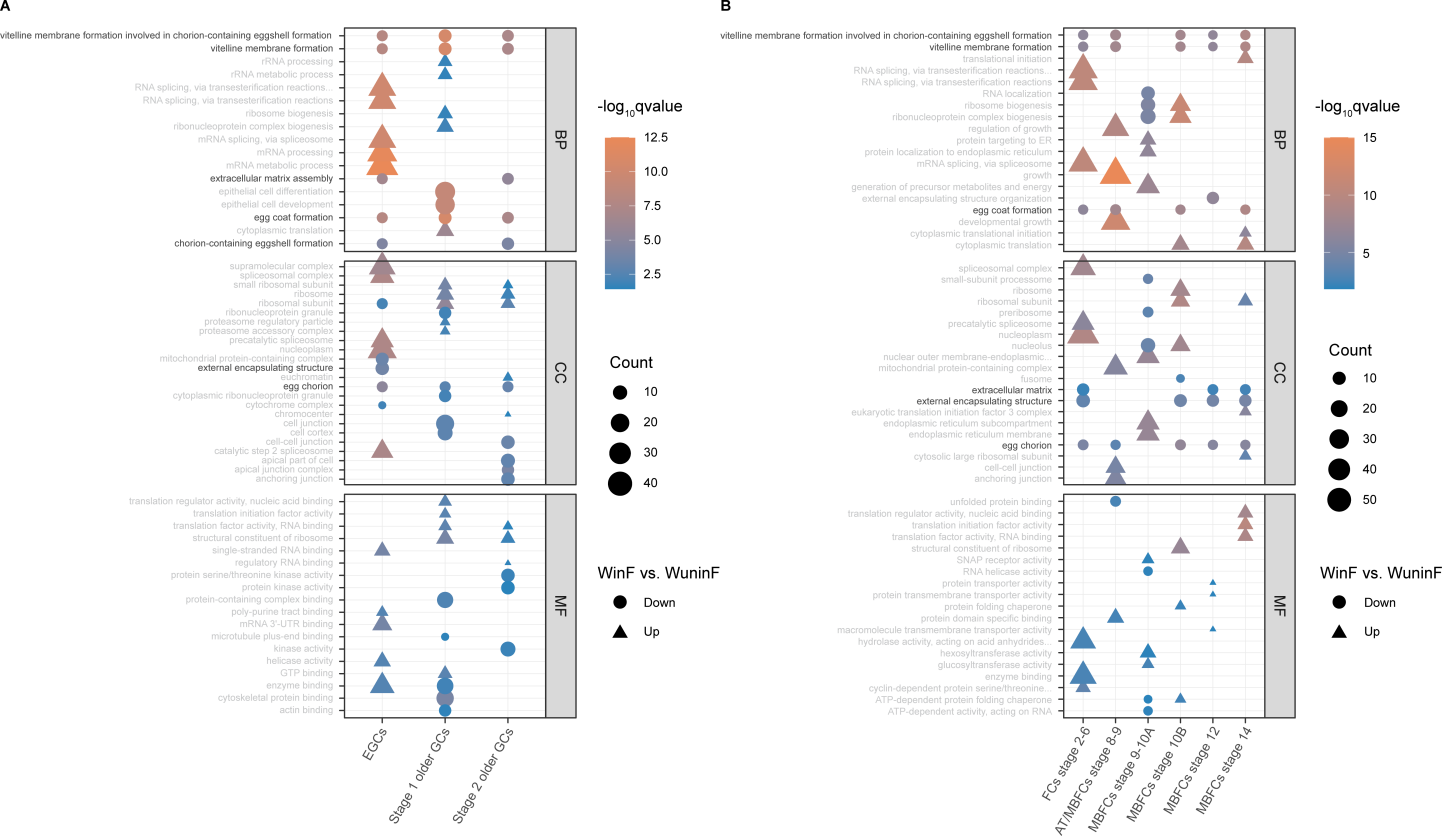
GO enrichment analysis of DEGs in germ cells and follicle cells. (**A**) GO enrichment analysis of DEGs in three clusters of germ cells. The figure showed the top five upregulated and downregulated terms in every GO function [biological process (BP), cellular component (CC), and molecular function (MF)] of each cluster. (**B**) GO enrichment analysis of DEGs in six clusters of follicle cells. The figure showed the top three upregulated and downregulated terms in every GO function of each cluster. All GO terms were ordered by *q*-value, with a threshold of <0.05. The “Count” represented the quantity of DEGs enriched in the GO term.

**Fig 3:**
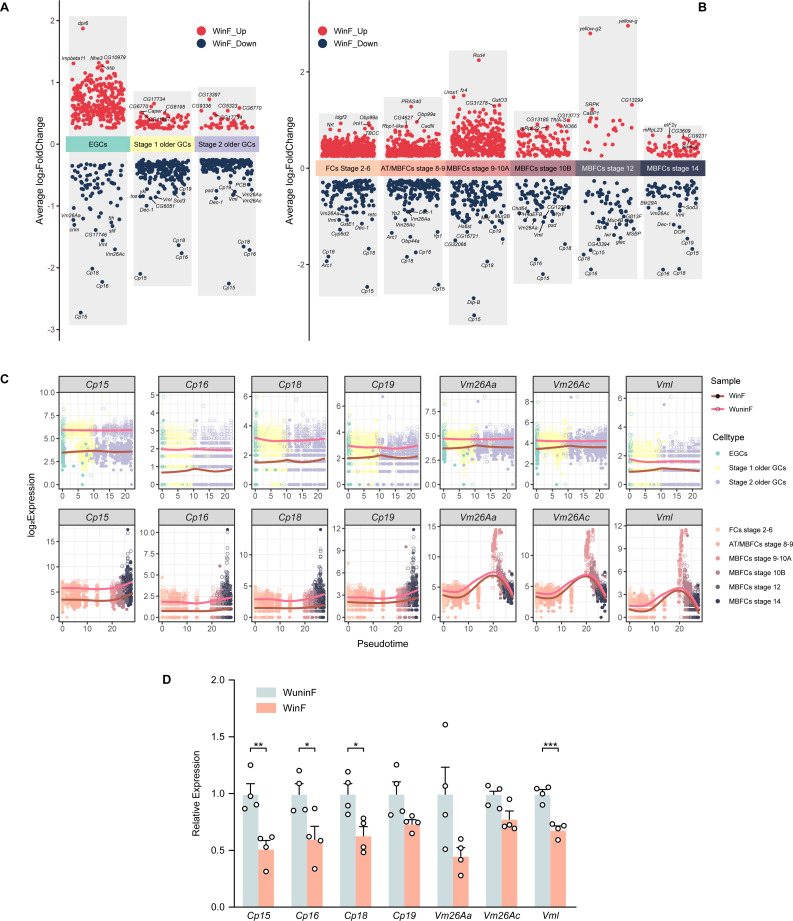
DEG expression patterns and certain DEG expression dynamics in germ and follicle cells. Volcano plots showed all DEGs identified in different development stages of germ cells (**A**) and follicle cells (**B**), and the parameters of DEG filtration were mentioned in the text content. The top five upregulated and top 10 downregulated DEGs were labeled in each cluster. (**C**) showed seven candidate DEGs that were significantly global-downregulated in multiple stages of germ and follicle cells. Smoothed curves described the expression dynamics of these genes over the psuedotime, which fitted by the method of loess. (**D**) RT-qRCR verification result showed that most candidate genes in (**C**) were significantly downregulated in WinF regarding overall ovarian levels. *P* value was calculated by Wilcox *t* test, and *P* value < 0.05 indicated statistical significance (*P < 0.05, **P < 0.01, ***P < 0.001).

In contrast to the diverse effects that *Wolbachia* exerts on different cell types of *Drosophila* testis we have reported (33), we here found some significant global effects on ovarian cell types caused by *Wolbachia* infection. Gene ontology (GO) enrichment analysis of DEGs (*P* value < 0.05) in the germ cells and follicle cells (that were the primary somatic cells) between WinF and WuninF was performed (Table S2). The top five GO terms of germ cells and top three terms of follicle cells were selected from the numerous results for presentation (Fig. 2). Interestingly, we discovered that the terms closely related to egg membrane formation were significantly downregulated in both germ and follicle cells of WinF (the term labels were highlighted in Fig. 2A and B), such as “vitelline membrane formation involved in chorion-containing eggshell formation” (GO:0007305) and “egg chorion” (GO: 0042600). Most of the genes in these terms were the top 10 significant downregulated genes among the DEGs, including *Vm26Aa*, *Vm26Ac*, *Vml*, *CP15*, *Cp16*, *CP18*, and *CP19* (Fig. 3A and B). *Vm26Aa*, also named *sV17*, is one of the three primary vitelline membrane structural proteins (the two others are *Vm26Ab* and *Vm32E*) (34–36), and *Vm26Ac* is often co-expressed with *Vm26Aa/b* (37). *Vml* can form a disulfide complex with *Vm26Aa* during the early stages of eggshell formation in the presence of *Vm26Ab* (38). Moreover, *Cp15/16/18/19* are all the crucial structural constituents of egg chorion. It is worth noting that the downregulation not only occurred widely from germline stem cells to the late-stage MB follicle cells of WinF (Fig. 3C) but was also identified in other cell types (Table S1).

We also performed RT-qPCR experiments on the above genes to verify their expression patterns based on the total ovarian RNA. Unsurprisingly, the mRNA transcription of *Cp15*, *Cp16*, *Cp18*, and *Vml* was significantly downregulated in WinF. Despite the absence of statistical significance in *Cp19*, *Vm26Aa,* and *Vm26Ac*, all the three genes also showed downregulation trends in WinF (Fig. 3D).

### Global effects of *Wolbachia* in cytoskeleton dynamics of ovarian cells

We continued to focus on the germ and follicle cell types and found that many genes enriched in the GO terms related to actin cytoskeleton organization were upregulated, and their numbers varied across five different cell types of WinF. For instance, the number of related upregulated genes was relatively higher in AT/MBFCs Stages 8–9 and PTFCs Stages 7–9 (44 and 40, respectively), while only three genes were identified in MBFCs Stage 14. Twenty related genes were upregulated in at least two different cell types, and eight of them were found in at least three cell types (Table S3).

Among the above eight genes, except for the four tubulin coding genes *βTub56D*, *βTub97EF*, *αTub84B*, and *αTub84D*, two genes involved in exocytosis, *Cdc42* and *Rab11*, aroused our interest. Rab11 is a crucial small GTPase that regulates endosomal recycling from the cytoplasm to the cytomembrane (39). It can form an exocyst complex with the Recycling Endosome (RE) and the protein Cdc42 (40), and the latter interacts with Moesin, which is located on the cytomembrane, to achieve the transport of RE (41). We also found upregulated genes enriched in the GO term of endocytosis in PTFCs Stages 7–9 of WinF, with *Rab5* being a representative gene (Table S3). Like Rab11, protein Rab5 forms a complex with Cdc42 and the Early Endosome (EE) during cytophagy. Our results indicated that *Wolbachia* infection possibly enhanced the frequency of exocytosis and endocytosis in follicle cells but not in germ cells, as *Cdc42*, *Rab11* and *Rab5* were all upregulated in follicle cells, and *Cdc42* was the only one found in EGCs (Table S3). Besides, the gene *trio*, encoding a Rho guanine nucleotide exchange factor (GEF) that activates the Rho-family GTPases, was upregulated in both EGCs and AT/MBFCs Stages 8–9. Trio acts within the Abl tyrosine kinase signaling pathway to regulate the dynamics of branched actin networks (42), thereby controlling cell motility and adhesion (43). Interestingly, the two upregulated Rho-family GTPases we identified in multiple cell types, *Cdc42* and *Rho1*, are both regulated by *trio* (44, 45). Although we did not observe a significant expression change in the gene *Abl* which acts upstream of *trio*, the substrate and positive regulator of Abl (*Abi*) (46) was upregulated in follicle cells. All evidence suggested that *Wolbachia* might influence the signaling pathway of actin cytoskeleton dynamics from upstream and regulate the motility of the host cytomembrane.

**Fig 4:**
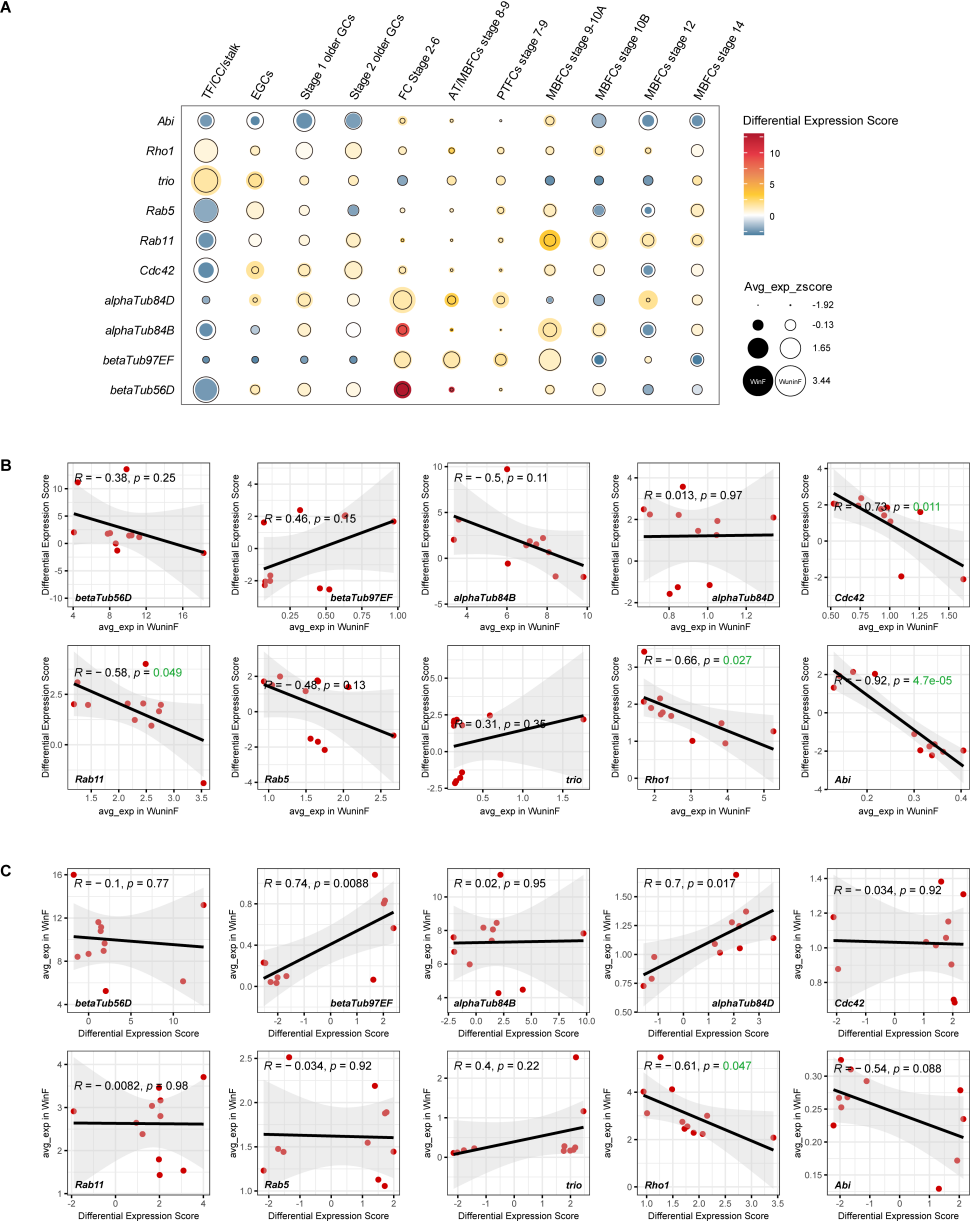
Correlations between the average expression level and the expression difference level of genes involved in cytoskeleton dynamics. The heatmap (**A**) displayed the average expression level in WinF and WuninF, and the expression difference level of 10 genes. For a more intuitive presentation, we scaled the average expressions among 11 cell types of each gene using *z*-score. Dotplot (**B**) illustrated the correlation between the average expression level in WuninF and the expression difference level of 10 genes and (**C**) showed the correlation between the average expression level in WuninF and the expression difference level of 10 genes. Spearman correlation coefficient was used for the correlation analyses. The 95% confidence intervals were highlighted in light gray, *P* value < 0.05 indicated statistical significance and was colored in green. Differential Expression Score (DES) was introduced to present the expression difference level. $DES=log_{10}^{\left(\frac{\left|log_{2}^{FoldChange}/p.adjust\right|}{\left|min\left(log_{2}^{FoldChange}/p.adjust\right)\right|}\right)}$. Larger value of DES indicates more significant and more intense differential expression of the gene, and the minus sign added before the DES represented the gene was downregulated.

Furthermore, we aimed to observe the correlation between the average expression level and the expression difference level of each gene we mentioned above. Here, we obtained the expression differences of all genes between WinF and WuninF, regardless of the previous filters, like expression percentage or *P* value in cell types. Instead, we introduced a new statistical method to present the gene expression difference and its confidence level simultaneously, called Differential Expression Score (DES) (Fig. 4). We found an unusual phenomenon that some genes trended to be downregulated in the cell types where it was highly expressed in WinF and vice versa. This phenomenon was evident in the gene *Abi* (Fig. 4A), which led us to hypothesize that the expression difference level of these genes was related to the average expression level before *Wolbachia* infection. As expected, there were significant negative correlations between the DES and the average expression level of genes *Cdc42*, *Rab11*, *Rho1*, and *Abi* in WuninF. Genes *βTub56D*, *αTub84B*, and *Rab5* showed the same trend despite having no significance (Fig. 4B). However, little apparent correlation was identified between the DES and the average expression level of the above genes in WinF, except *Rho1*, indicating that the final effect of *Wolbachia* infection on host genes’ expression is indeterminate.

### Specific effects of *Wolbachia* in the germ cells

Finally, we characterized two specific effects of *Wolbachia* on germ cell types in WinF. First, a group of DEGs enriched in the GO terms associated with ribonucleoprotein complex biogenesis was upregulated in EGCs of WinF, including 20 members, but few of these genes were significantly altered in other cell types, except for only four genes upregulated in MBFCs Stage 10B. In addition, the cell typing marker gene for Stage 1 older GCs, *orb*, was abnormally downregulated in this cell type. Second, 30 upregulated genes in EGCs of WinF were specifically enriched in the GO terms associated with cell proliferation (such as “mitotic cell cycle” or “cell population proliferation”) (Table S4). However, 12 downregulated genes involved in cell proliferation were found in Stage 1 older GCs, while only one of these was a member of the above 30 genes in EGCs. We observed the DES pattern of these genes in germ and follicle cell types, similar to the analysis of genes related to cytoskeleton dynamics. The result was almost consistent with the strict filtration of DEGs (Fig. S1A). Gene set enrichment analysis also showed that the signaling pathways related to cell cycle and ribonucleoprotein complex biogenesis tended to be upregulated in EGCs, but without significance (Fig. S1B). These results suggested that the growth and division of germ cells were affected, particularly enhanced in EGCs by *Wolbachia*.

## DISCUSSION

In this study, we identified and characterized multiple cell types in the ovary through single-cell RNA sequencing of ovaries from *D. melanogaster* and explored the effects of *Wolbachia* infection on each cell type in gene expression. We aimed to investigate how *Wolbachia* promotes its spatial transmission within ovarian cells and localization to the oocyte, and to discuss the potential mechanism through which *Wolbachia* promotes its own maternal inheritance in the host ovaries.

### Extracellular

*Wolbachia* infection caused significant downregulation of multiple genes encoding vitelline membrane and chorion membrane structure proteins in ovarian cells. Considering that the multilayered structure of the *Drosophila* eggshell is an ideal experimental system for studying extracellular matrix formation *in vivo* (38), eggshell-related proteins may play similar roles in the extracellular environment of the other cell types. Therefore, we speculated that the global downregulation of these genes led to a loose extracellular matrix structure of the germarium, thus potentially reducing the obstacle that *Wolbachia* will encounter during transmission across ovarian cells. However, it seems that the reductions of egg membrane proteins do not substantially harm the mature egg production. This possibility is supported by the fact that the increased egg production of *Drosophila mauritiana* infected with *Wolbachia* strain *w*Mau has been verified (8).

### Across membrane

The horizontal transfer of *Wolbachia* from cell to cell involves a host cell phagocytic and Clathrin/dynamin-dependent endocytic mechanism (47), and dynamin is a class of GTPases responsible for the formation and release of vesicles. Although we did not find any changes in transcription levels of Clathrin in *Drosophila* ovaries, significant upregulation of GTPases *Rab5*, *Rab11*, and *Cdc42*, which are closely related to vesicle transport during phagocytosis and exocytosis, occurred in multiple cell types. The upregulation of *Rab11* not only simply enhanced the frequency of exocytosis but also was positively correlated with phagosome formation through focal exocytic activity (40, 48). It is interesting that when we loosened the filters for DEGs and focused on the trend of genes’ differential expression, we discovered significant negative correlations between DES and the average expression level of genes involved in cytoskeleton dynamics, such as *Cdc42*, *Rab11*, *Rho1*, and *Abi*, which means that *Wolbachia* employs a flexible regulation strategy to affect the motility of the host cytomembrane. *Wolbachia* has the capacity to upregulate or downregulate the expression level of specific host genes according to its own needs but not rigidly change the expression level in one direction to create a more suitable living environment in the host’s ovarian cells. This explains the phenomenon mentioned above that the gene *orb* specifically underwent repression in Stage 1 older GCs due to its initial high expression level in this cell type. All these results indicate that *Wolbachia* affects the phagocytosis in the host ovarian cells, which is beneficial for its horizontal transmission from one cell to another. Additionally, among the DEGs we described above, *trio* was an essential gene that acted with *Abi*, *Rho1* and *Cdc42*, and *Cdc42* was closely associated with *Rab5* and *Rab11* for the control of cytoskeleton dynamics, which made us speculate that *Wolbachia* might influence the host through cascade regulations by directly affecting few upstream core genes of the signaling pathways.

### Intracellular

Actin and cytoskeleton are also essential for the subcellular localization of *Wolbachia* within *Drosophila* cells. For example, *Wolbachia* can utilize microtubules and dynein to locate into the host oocytes at an early stage (49) or produce a broad but uneven distribution pattern in diverse tissues during embryonic development (50). Thus, the numerous upregulated genes related to cytoskeleton synthesis, like the four tubulin coding genes in multiple cell types of WinF, implies that *Wolbachia* could facilitate cytoskeleton formation and consequently be beneficial to its localization and spread in the host ovarian cells.

In addition, *Wolbachia* secretes the TomO protein to interact with mRNAs encoding Nanos and Orb proteins in *Drosophila* (51, 52). These mRNAs are not translated until they are carried by ribonucleoprotein-complex processing bodies (P-bodies) and transported through microtubules to the posterior pole of the oocyte (53). *Wolbachia* is often observed in the vicinity of P-bodies in female germ cells, implying that it may be directed into the oocyte through ribonucleoprotein-complex transport (54). In the EGCs of WinF, *nos* and *orb*, the genes encoding Nanos and Orb protein, respectively, were both globally upregulated. Although we could not prove that the two proteins are produced more by *Wolbachia* infection due to lacking evidence of proteome, there is no doubt that the enhanced gene transcription in both GSCs and cyst increases the possibility of its localization to the host oocytes through binding with P-bodies. This is because it guarantees sufficient mRNAs of *nos* and *orb* in the oocyte no matter which cell in the cyst differentiates into the oocyte. Previous reports have shown a significant accumulation of *Wolbachia* in the host oocytes from Stage 2 (even much earlier) (5, 10), which is consistent with our results. This controlled accumulation ensured a sufficient titer of *Wolbachia* in mature eggs, which benefited *Wolbachia*’s maternal transmission.

### Conclusion

In summary, we have discussed a series of potential effects of *Wolbachia* on the host ovary from extracellular to intracellular, through which we speculate *Wolbachia* promotes its spatial transmission within host ovarian cells and localization to the oocyte. The unique aspect of this study is that we are mainly concerned with the global gene differential expressions among multiple cell types rather than finding specific gene expression patterns in each cell type, as most of the studies employing single-cell RNA sequencing have done, because we hypothesize that a simple global-scale effect on the host is more easily achieved, especially for an endosymbiont like *Wolbachia*. The results demonstrate that many of the global effects of *Wolbachia* on host ovarian cells are concentrated on the transcription of structural material proteins or upstream of signaling pathways, and the transcription of genes related to ribonucleoprotein complex biogenesis in early germline cells is also affected, both of which can well support our hypothesis. However, a challenge of this study is that we observed several gene expression changes but have yet to determine the core genes directly affected by *Wolbachia* using convincing experimental validation, let alone discovering the *Wolbachia*-derived factors. Moreover, it is surprising that *Wolbachia*’s regulatory influence on the host is possibly bi-directional, which leads us to hypothesize further that there may be a feedback regulatory system between *Wolbachia* and its host. Therefore, the search for molecular evidence of interactions or feedback regulatory systems between *Wolbachia* and host ovaries will be a major focus of future studies.

## MATERIALS AND METHODS

### Preparation of single-cell suspension and tissue dissociation

*Wolbachia* (strain *w*Mel)-infected and uninfected *Drosophila* strains were established following our previously described method (26) and reared for more than 40 generations before the beginning of this study. The infected and uninfected virgin females (3-day-old) were used in this study. We anesthetized 20–30 females on ice and dissected them in cold Earle’s balanced salt solution (EBSS) (NaCl 6.8 g/L, NaH_2_PO_4_ 0.112 g/L, KCl 0.4 g/L, d-glucose 1 g/L, NaHCO_3_ 2.2 g/L without calcium, magnesium, and phenol red). Dissected ovarian tissue was temporarily stored in cold insect medium (Grace’s insect basal medium + 15% fetal bovine serum) to maintain optimal cell viability, and the entire dissection process was completed within 30 min. Next, we removed the insect medium and added 200 µL of lysis solution (EBSS containing 4 mg/mL elastase and 2.5 mg/mL type IV collagenase) for ovarian tissue lysis at room temperature (25°C) for 30 min. The cell suspension was passed through a 40-µm cell sieve and collected in a low-adsorption centrifuge tube. After centrifugation at 4°C and 300 × *g* for 5 min, the cells were re-suspended in 1 mL of cold EBSS and centrifuged at 4°C and 300 × *g* for 5 min. The supernatant was removed, and the cells were re-suspended in 200 µL of cold EBSS. Cell viability was detected using Typan blue stain solution (Solarbio, China). Finally, the single-cell suspension was diluted to approximately 1,000 cells/µL, and the libraries were constructed with the 10× Genomics chromium 3′ kit and sequenced on MGISEQ2000 (BGI, Shenzhen, China) instruments using 100 nt paired-end, with at least 7 million reads per library.

### Differential gene expression analysis

The FASTQ file of each sample was used for scRNA-seq downstream analysis. The reference genome and annotation file (GTF) of *D. melanogaster* were downloaded from Ensembl Genome Browser (Drosophila_melanogaster.BDGP6.32.dna.toplevel.fa and Drosophila_melanogaster.BDGP6.32.108.gtf). The annotation file was filtered using the “mkgtf” command of CellRanger (v7.1; https://support.10xgenomics.com/singlecell-gene-expression/software/downloads/latest) with the parameter “attribute = gene_biotype: protein_coding.” Genome and filtered annotation were then used as input to the “mkref” command to build an appropriate CellRanger Reference. The output files were imported into the Seurat (v4.4.0) R package (55) for quality control and downstream analysis. We filtered out cells with ≤200 genes, ≥6,000 genes, or ≥5% mitochondrial genes. Following the official guide of Seurat (56), we used SCTransform workflow in Seurat with the parameter of vars.to.regress = “percent.mt” to eliminate the interference from the mitochondrial genes. DoubletFinder software (57) was adopted for doublet identification and possible doublet removal from the scRNA-seq data. Then we used the “FindClusters” command in Seurat, set the resolution to 0.9 to cluster all cells, and ran the UMAP function in Seurat with default parameters. The cells were clustered based on the first 14 principal components, and the specific markers in each cluster were identified by the “FindAllMarkers” command (options: only.pos = TRUE, min.pct = 0.5, and logfc.threshold = 0.25). We used the reported marker genes in previous studies for cell type identification. We identified DEGs for each cell type between infected and uninfected samples using the “FindMarkers” command in Seurat (logfc.threshold = 0.25, min.pct = 0.25, and test.use = Wilcox), and *P* value < 0.05 was considered statistically significant.

### Functional enrichment analysis

For DEGs, gene ontology enrichment analysis was performed using R package clusterProfiler (version 4.8.3) (58) with Fisher’s exact tests (two-sided), and the *P* value was adjusted using the Benjamini-Hochberg (BH) procedure. The adjusted *P* value (*P*_adj)  < 0.05 indicated statistical significance.

### Pseudotime inference analysis and DEGs expression dynamic over pseudotime

The R package Monocle3 (v1.3.4) (59) was used to analyze single-cell pseudotime trajectories and reveal DEG expression patterns, and “UMAP” was applied to reduce dimensions. Germ cell and follicle cell clusters were selected separately to construct the pseudotime, and on this basis, the changes of DEGs between WinF and WuninF with the pseudotime were observed.

### RT-qPCR experiment for verification of certain DEGs expression

To verify the expression patterns of the DEGs we were concerned with, we dissected female fruit flies (3-day-old) and acquired the ovaries using the same method as above. Following the instructions of *TransZol* Up Plus RNA Kit (TransGen Biotech, China), we extracted 15 ovaries’ total RNA as a single sample and four samples of each of WinF and WuninF were prepared. Then we used EasyScript All-in-One First-Strand cDNA Synthesis SuperMix (TransGen Biotech, China) to obtain the cDNA of each sample for subsequent qPCR experiments. We used the *β-spec* gene as an internal control when performing qPCR experiments. The reaction mix of qPCR was prepared using *PerfectStart* Green qPCR SuperMix (TransGen Biotech, China), and the primers used in this study are listed in Table S5.

### Statistical analysis

All statistical analyses were performed using R packages or Minitab Statistical Software (v21.1). Statistical analysis of functional enrichment was performed using Fisher’s exact test, and the *P* value was adjusted using the Benjamini-Hochberg method. The adjusted *P* value < 0.05 indicated statistical significance. Statistical analysis of gene expression verification was performed using the Wilcox *t* test, and a *P* value  <  0.05 indicated statistical significance.

## Data Availability

The raw data of scRNA-sequencing used in this study were uploaded to the following link: https://www.ncbi.nlm.nih.gov/sra/PRJNA1108780

## SUPPLEMENTAL MATERIAL

Fig. S1 mbio.01473-24-s0001.docx Genes specifically changed in germ cell types.

Table S1 mbio.01473-24-s0002.xlsx DEGs in *Drosophila* ovarian cells (WinF vs WuninF).

Table S2 mbio.01473-24-s0003.xlsx The GO enrichment of DEGs in germ cells and MB follicle cells.

Table S3 mbio.01473-24-s0004.xlsx GO terms related to actin cytoskeleton organization.

Table S4 mbio.01473-24-s0005.xlsx GO terms related to ribonucleoprotein complex biogenesis and cell proliferation in germ cells.

Table S5 mbio.01473-24-s0006.xlsx Primers for RT-qPCR in this study.

ASM does not own the copyrights to Supplemental Material that may be linked to, or accessed through, an article. The authors have granted ASM a non-exclusive, world-wide license to publish the Supplemental Material files. Please contact the corresponding author directly for reuse.
