# Supplementary material for: Single-cell transcriptome sequencing reveals that Wolbachia induces gene expression changes in Drosophila ovary cells to favor its own maternal transmission
Source: mBio. 2024 Aug 28;15(10):e01473-24. doi: 10.1128/mbio.01473-24 (PMC11481584; doi:10.1128/mbio.01473-24)
Supplement: Fig. S1 — Genes specifically changed in germ cell types. [file mbio.01473-24-s0001.docx]

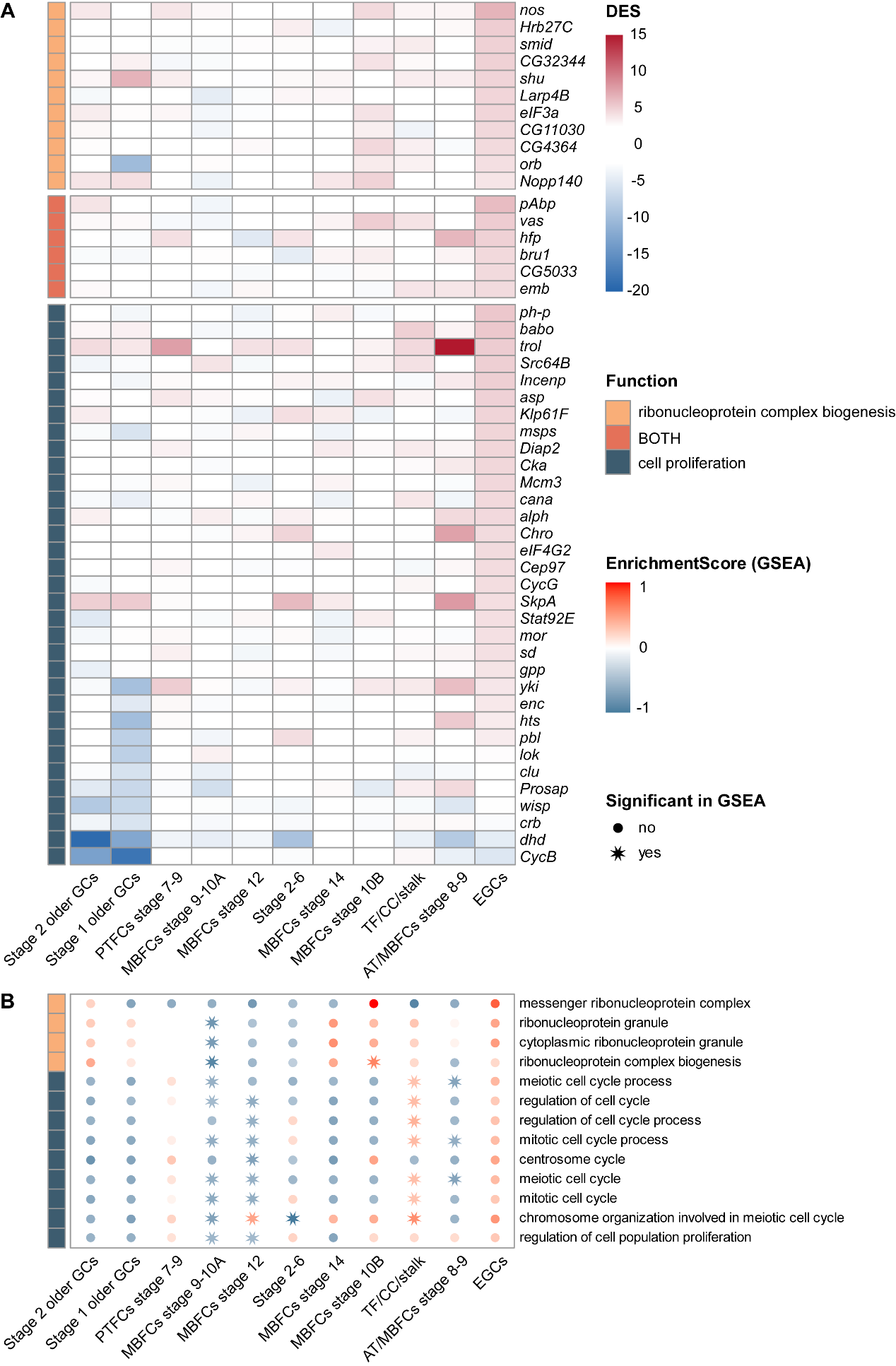


**Fig. S1 Genes specifically changed in germ cell types.** (A) The heatmap presented expression trends of the significantly regulated genes related to ribonucleoprotein complex biogenesis and cell cycle in germ cells. Most genes showed upregulation trends in the EGCs of WinF, while 13 genes were downregulated in Stage 1 older GCs or Stage 2 older GCs. (B) The dot plot presented GSEA analyses of GO terms involved in ribonucleoprotein complex biogenesis and cell cycle in each cell type. *p*-value < 0.05 and *q*-value < 0.25 indicated statistical significance, and the *p*-value was corrected by BH method.
